# No Clear Effect of Admixture between Two European Invading Outbreaks of *Diabrotica virgifera virgifera* in Natura

**DOI:** 10.1371/journal.pone.0106139

**Published:** 2014-08-29

**Authors:** Gérald Bermond, Fanny Cavigliasso, Sophie Mallez, Joseph Spencer, Thomas Guillemaud

**Affiliations:** 1 UMR 1355 Institut Sophia Agrobiotech, INRA, Sophia Antipolis, France; 2 UMR Institut Sophia Agrobiotech, Université de Nice-Sophia Antipolis, Sophia Antipolis, France; 3 UMR 7254 Institut Sophia Agrobiotech, CNRS, Sophia Antipolis, France; 4 Illinois Natural History Survey, University of Illinois, Champaign, Illinois, United States of America; Fordham University, United States of America

## Abstract

In this study, we challenged the hypothesis that admixture may have had a positive impact in the context of the European invasion of the western corn rootworm (WCR), *Diabrotica virgifera virgifera*, LeConte. This beetle was introduced in Europe from the USA several times since the 1980’s. The multiple introductions of this major pest of cultivated corn led to the formation of two major outbreaks in North Western (NW) Italy and in Central and South Eastern (CSE) Europe that eventually merged into a secondary contact zone where insects from both outbreaks interbreed. We collected about 600 insects from this contact zone and genotyped them using 13 microsatellite markers. Three types of information were obtained from the collected individuals: (i) their survival under starvation; (ii) their admixed status, determined through a Bayesian method of genetic clustering and (iii) their mating probability, studied *via* the detection, isolation and genotyping of sperm in female spermathecae. Twenty six % and 12% of the individuals were assigned to the NW Italy or the CSE Europe parental types, respectively, and 23% and 39% to the F1 and backcross hybrid types, respectively. Globally, our results do not reveal any significant impact of the admixed status on the mating probability and on the choice of mating partners. However the admixed status had a sex- and sampling site-dependent effect on survival in adults under starvation. In addition sex had an effect on survival, with mortality hazard about 3 times larger in males than in females. The consequences of these findings for the evolution of the admixture zone of northern Italy are discussed.

## Introduction

Despite the increase of the number of invasive species, only a small fraction of emigration events leads to successful introductions, settlements and proliferations into new territories [1]. To better control or simply avoid the colonization of new territories by exotic species, biologists have tried for sixty years to investigate and understand the complex ecological processes involved in invasions [2], [3]. According to Fauvergue et al [4], three factors can explain the success or failure of introductions. First, abiotic and biotic environmental conditions of the new area may be unfavorable for the introduced species, e.g. [5]. Secondly, the explanation can also be demographic: bottlenecks experienced during introductions generate other processes such as demographic stochasticity [6] and Allee effects [7] that can lead to the extinction of the introduced populations. Finally genetics can also be involved: population introductions are often associated with founder events that are generally associated with a loss of genetic diversity that can lead to a reduction in the adaptive potential of populations [8]. The consequences of this reduction may be important for the introduced populations since the invaded environment presents new selection pressures that will act on a limited range of genetic variability. Bottlenecks can also induce an increase of consanguinity in introduced populations, generate inbreeding depression and even the fixation of deleterious alleles. Such processes will increase the probability of extinction of introduced populations [4].

During biological invasions, the loss of genetic diversity associated with bottlenecks [9], [10] is sometimes offset in the invaded area by intraspecific hybridization (*i.e.* admixture) between populations that have been introduced from several source populations from the native area [11]. These multiple introductions from different population sources are common [12] and sometimes lead to the formation of a single outbreak in which admixture occurs [13]. Such introductions can also lead to the formation of several outbreaks that are geographically disconnected [14]–[18] and genetically differentiated [19]. Thereafter, the expansion of these outbreaks can then lead to a secondary contact, e.g. [20], where populations meet, cross and form an admixed zone, e.g. [21].

Such admixture can have beneficial consequences for the invasive populations [11], [22] and promote adaptation to new environments, e.g. [14], [23]. First, the admixture can increase the extent of genetic variation displayed by populations [15] and allow selection to operate on a broader range of genetic variability. Second, recombination between genotypes from genetically differentiated populations can create new genotypes and increase the phenotypic range on which the natural selection will act, e.g. [24], [25]. Finally, admixture can also lead to heterosis – *i.e.* a larger fitness of the admixed individuals than that of the parental individuals [26], [27]. This may occur when the parental introduced populations have fixed a set of different recessive deleterious mutations that become heterozygous after the cross of the populations [28]. Such theoretical predictions have been confirmed in various species like flowering plants [29], freshwater snails [30], and crops [31]. Although other mechanisms such as overdominance exist to explain heterosis, their effect is generally considered less important than the masking of genetic load [11].

Admixture is thus often considered as a stimulating effect of successful invasions [22], [32]. However, some authors have suggested that the stimulatory effect of admixture during invasions was often over-estimated, e.g. [33]. Admixture can lead to outbreeding depression, which is a decrease in the fitness of admixed individuals relative to their parents [34], [35]. This can happen through (i) underdominance [36], (ii) the breaking down of co-adapted gene complexes [37] and (iii) genetic incompatibilities of Dobzhansky-Muller type [38], *i.e.* the whole of independent genetic changes that occur in isolated populations and that must be compatible with their own genetic backgrounds but need not be compatible with other genetic backgrounds [39].

Here, we used the western corn rootworm (*Diabrotica virgifera virgifera* LeConte, WCR) as a biological model to study the impact of admixture in an invasion context. This univoltine beetle (Family Chrysomelidae), native to Central America, is one of the most damaging pests of corn in the USA and its associated damages exceed US $1billion per year [40]–[42]. Several introductions of WCR from the USA have succeeded in Europe [19], [43] since the late 1980’s. These introductions led to the formation of two genetically differentiated main outbreaks (with a mean *F*_ST_ of 0.26 [46]): the outbreak of Northwestern Italy (NW Italy), first observed in 2000, and the Central and South Eastern Europe (CSE Europe) outbreak, first observed in 1992 [19]. From 2004, these two outbreaks converged on each other and came into contact in 2008 in the Veneto region of Northern Italy, thus forming an admixed zone [21]. Bermond *et al* demonstrated the presence of only two parental populations in this particular case of admixture [21], [44]. In this study, we collected 600 adult individuals at the centre of the admixed zone and we genotyped them using 13 microsatellite markers. Three types of information were obtained from the collected individuals: (i) their survival under starvation; (ii) their admixed status, determined through a Bayesian method of genetic clustering, and (iii) their mating probability, studied *via* the observation, isolation and genotyping of sperm in female spermathecae. We statistically tested for a link between the individual admixed status and a) mating probability and b) individual survival under starvation. Finally we tested the hypothesis of assortative mating in the contact area, *i.e*. if there is preferential mating between individuals of certain admixed status.

## Materials and Methods

### Sample collection and survival measures

**Figure 1:**
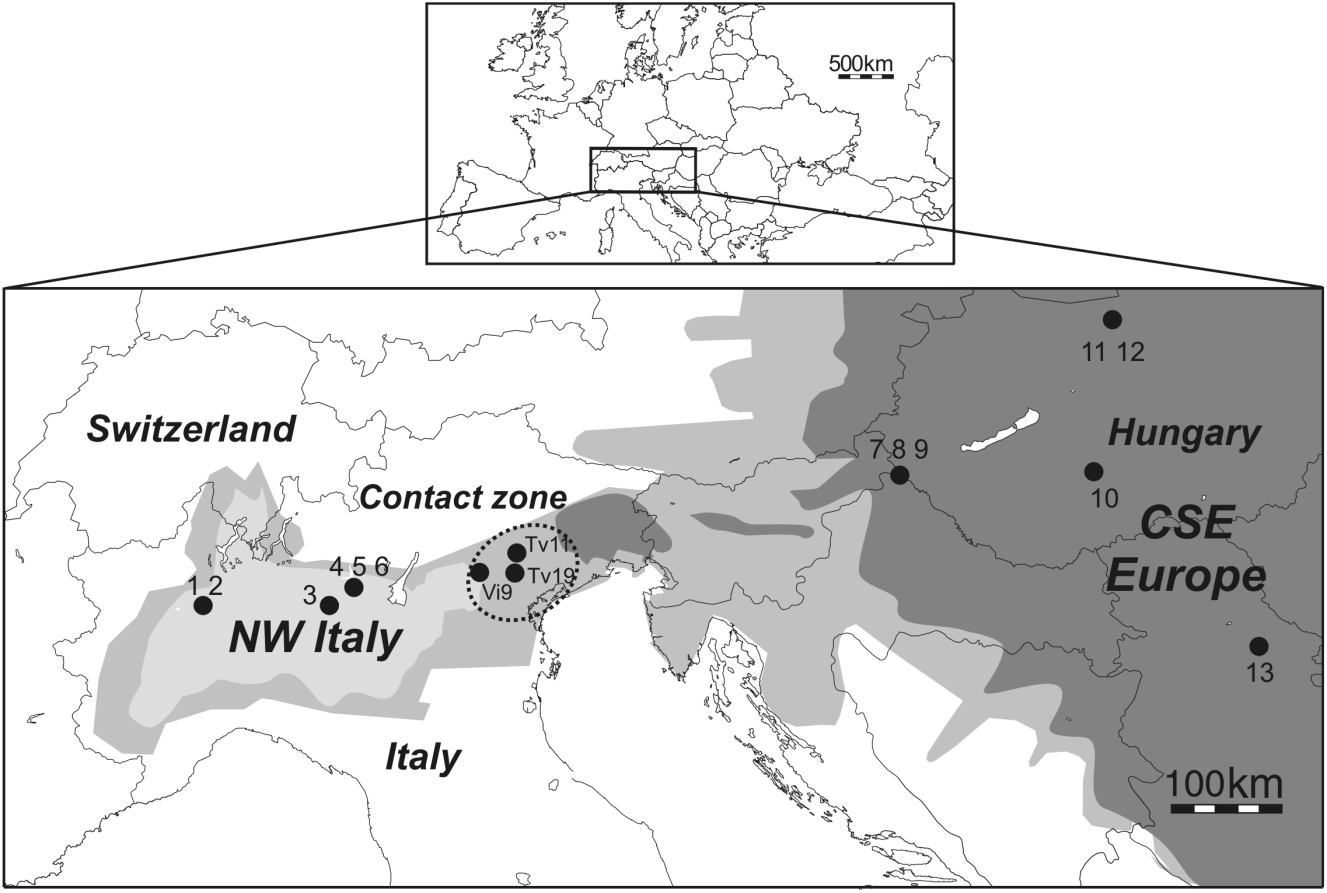
Location of the main expanding outbreaks of *Diabrotica virgifera virgifera* in Europe (North West (NW) Italy and Central South Eastern (CSE) Europe) and location of samples collected in 2012 in the contact zone (Veneto, Northern Italy), represented by the dotted circle. Numbers refer to sample names (see Table 1). The distribution areas of WCR before contact between the western and eastern outbreak populations (in 2007) are shown in light gray (NW Italy) and dark gray (CSE Europe). The distribution area of 2012 (after contact) is shown in medium gray.

We selected 3 fields located in the center of the admixed zone (see Figure 1) to maximize the likelihood of sampling all genotypic types (West parental, East parental and admixed) in close geographic proximity to minimize the environmental component of the phenotypic variance.

No permission is required to collect samples of this species at any location. The study did not involve an endangered or protected species. WCR adults were sampled in three locations in the province of Vicenza (Vi9) and Treviso (Tv11 and Tv19), during summer 2012, between the 19^th^ and the 20^th^ of July in the region of Veneto, in northern Italy (Figure 1; see details in Table 1). GPS coordinates of sampling locations are provided in Table 1. For each sampling site, 200 WCR adults were collected from a single cornfield. Sampling was conducted to obtain a roughly balanced sex-ratio (see details in Table 2). Adult beetles were caught by hand or by using a mouth vacuum and were then placed individually without food in ventilated (perforated) flat-top 1.5-mL polypropylene microtubes. Microtubes were stored in open plastic storage boxes and held under controlled conditions in climatic rooms at 23°C with a 16∶8 (L:D) photoperiod and 50–80% RH (respectively for day and night) until all individuals had died. For each collected adult, we measured the survival time under starvation conditions (without food and water). We counted the number of dead individuals per sample site twice a day, late in the morning (11 am) and in the evening (8 pm). The remaining living adults within each storage box were randomly repositioned at each counting to avoid any effect of location in the box on survival. After death, individuals were stored in 96% ethanol at 4°C.

**Table 1:** Description of the within-population genetic variation of *Diabrotica virgifera virgifera* samples from Veneto, North-West Italy and Central and South-Eastern Europe.

Type of sample	Sample number	Sample name	Outbreak	Region/Country	Sampling date	*A*		*He*	*F_IS_*	*p-HW*	*N*	GPS coordinates (Latitude; Longitude)
						*DC*	*AR*					
Principal	-	Tv11	Contact zone	Veneto/Italy	2012	4.92 (3.73)	3.72 (2.37)	0.48	0.08	<10^−3^	252	(45°47′52.80″N; 11°53′33.50″E)
	-	Tv19	Contact zone	Veneto/Italy	2012	4.46 (3.10)	3.56 (2.20)	0.47	0.05	<0.05	268	(45°43′26.69″N; 11°52′57.61″E)
	-	Vi9	Contact zone	Veneto/Italy	2012	4.85 (3.58)	3.58 (2.30)	0.48	0.05	0.01	260	(45°33′58.70″N; 11°24′59.40″E)
Reference	1	Olcenengo	NW Italy	Piedmont/Italy	2011	3.77 (2.42)	3.62 (2.32)	0.43	−0.08	0.64	30	(45°21′59.50″N; 08°18′18.40″E)
	2	Olcenengo	NW Italy	Piedmont/Italy	2012	4.00 (2.58)	3.76 (2.52)	0.41	−0.04	0.17	35	(45°21′59.50″N; 08°18′18.40″E)
	3	Fontanella	NW Italy	Lombardia/Italy	2007	2.77 (1.79)	2.67 (1.69)	0.37	0.06	0.54	33	(45°26′00.40″N; 9° 47′48.54″E)
	4	Castegnato	NW Italy	Lombardia/Italy	2010	3.00 (1.92)	2.89 (1.79)	0.37	−0.06	0.54	30	(45°34′06.52″N; 10° 6′35.63″E)
	5	Castegnato	NW Italy	Lombardia/Italy	2011	3.08 (1.80)	2.95 (1.68)	0.38	0.00	0.95	30	(45°34′06.52″N; 10° 6′35.63″E)
	6	Castegnato	NW Italy	Lombardia/Italy	2012	3.08 (2.18)	2.87 (1.89)	0.39	0.00	<10^−3^	40	(45°34′06.52″N; 10° 6′35.63″E)
	7	Pince	CSE Europe	Prekmurje/Slovenia	2010	2.77 (1.24)	2.77 (1.23)	0.44	0.08	0.06	27	(46°31′15.38″N; 16°31′23.70″E)
	8	Pince	CSE Europe	Prekmurje/Slovenia	2011	2.85 (1.35)	2.83 (1.32)	0.45	0.05	0.74	31	(46°31′15.38″N; 16°31′23.70″E)
	9	Pince	CSE Europe	Prekmurje/Slovenia	2012	3.00 (1.41)	2.87 (1.28)	0.45	0.02	0.79	41	(46°31′15.38″N; 16°31′23.70″E)
	10	Szekszard	CSE Europe	Tolna/Hungary	2007	2.77 (1.24)	2.76 (1.23)	0.46	−0.05	0.98	39	(46°30′59.52″N; 18°37′57.72″E)
	11	Budapest	CSE Europe	Pest/Hungary	2011	2.77 (1.24)	2.77 (1.24)	0.43	0.02	0.68	30	(47°31′12.90″N; 18°46′33.10″E)
	12	Budapest	CSE Europe	Pest/Hungary	2012	2.85 (1.57)	2.75 (1.39)	0.44	0.07	0.30	40	(47°31′12.90″N; 18°46′33.10″E)
	13	Crepaja	CSE Europe	Voïvodine/Serbia	2007	2.92 (1.50)	2.89 (1.43)	0.46	0.00	0.59	30	(45°2′55.38″N; 20°38′23.52″E)

**Note:** The column “Type of sample” describes the different samples used in this study: “Principal” indicate samples on which phenotypic traits and clustering analyses were respectively measured and conducted whereas “Reference” indicate samples that were only used in the clustering analyses. NW: North-West. CSE: Central and South-Eastern. *N*: sample size. *A*: mean number of alleles per locus. *A* was determined by direct counts (*DC*) and allelic richness (*AR*) analysis. *AR* is based on the smallest sample size (*N* = 24 for one locus of the sample Budapest 2011). Standard deviations between loci are shown in parentheses. *He*: mean expected heterozygosity [46]. *p-HW*: p-values for the exact test of deviation from *HW* equilibrium.

**Table 2:** Description of the *Diabrotica virgifera virgifera* samples from the Veneto contact zone, in Northern Italy.

Sample name	Italian Province/Region		Females			Males		
		*N*	No of sampled	No of genotyped	No of mated among genotyped	No of sampled	No of genotyped	No of genotyped spermathecae
Tv11	Treviso/Veneto	252	97	96	83	103	103	53
Tv19	Treviso/Veneto	268	122	118	113	78	78	72
Vi9	Vicenza/Veneto	260	108	105	100	92	90	65

*N* represents the total number of genotyped items (genotyped females and males and genotyped spermathecae) for each sample. The number of genotyped spermathecae represents the number of males that successfully mated and which multilocus genotypes were determined from spermathecae.

### Determination of sex, dissection of spermathecae and isolation of sperm

WCR sex was confirmed by dissecting all individuals to look for female or male reproductive organs. If present, a female’s spermatheca is easily isolable under a stereo-microscope by pulling on the pygidium of the insect with a pair of fine forceps. After isolation, we placed the spermatheca in a 10 µL droplet of 0.065% NaCl aqueous solution on a microscope slide. Observations were done under a compound microscope (Zeiss, Axiomager Z1) at 200X and 400X magnification to reveal the presence of spermatozoa (Figure S1A and S1B, respectively). We cut the spermatheca into two pieces and crushed it to expel sperm from the spermatheca. To completely isolate sperm, we covered the droplet with a coverslip and used the back of the forceps to gently tap the coverslip several times. Spermatozoa were then visible as a mass of hair-like threads extruding from fissures in the spermatheca. We then removed the coverslip and added 10 µL of NaCl to facilitate the manipulation of the sperm mass using forceps and/or a needle. The tough sheath of the spermatheca was then completely removed and the sperm were directly transferred with a P10 micropipette in a well of a 96 well-PCR plate for DNA extraction.

### Genotyping

DNA was extracted from spermatozoa using the commercial kit prepGeM (ZyGeM Ltd, Hamilton, New Zealand) according the manufacturer’s instructions with an elution volume of 20 µL. Adults (males and females) were washed three times in 0.065% NaCl before extraction to remove the excess of ethanol from the tissues. For all individuals, we extracted DNA from the thorax or half of whole WCR body cut lengthwise. The adults’ DNA extraction was performed with the DNeasy tissue kit (Qiagen, Hilden, Germany) following the manufacturer’s instructions and with an elution volume of 100 µL. Thirteen microsatellite loci of WCR [21] were amplified using three separate multiplex PCRs performed in a S1000 Thermal Cycler and were analyzed as described by Miller et al. [45]. DNA amplification consisted of 25 and 33 PCR cycles for individuals and sperm, respectively.

The absence of maternal contamination in the sperm genotypes was verified by checking that the sperm genotypes contained at most 2 alleles because WCR is diploid. We also compared the genotypes of sperm and those of the associated females. The genotypes of the sperm should not be strictly identical to that of the maternal parent. Note that this is a stringent condition because, particularly for frequent alleles, those genotypes may be identical even without contamination.

### Data analysis

#### Genetic composition of samples

The genetic composition of the samples was determined by estimating summary population genetic statistics (see Table 1). Genetic variation within samples was evaluated by determining the mean number of alleles per locus (*A*) and mean expected heterozygosity (*He*) [46]. *A* and *He* were calculated with GENECLASS version 2.0.h [47]. We calculated the Weir and Cockerham [48] estimate of *F_IS_* with GENEPOP ver. 4.0.1 [49], [50]. We also computed the allelic richness (*AR*) based on the smallest sample size, by the rarefaction method [51] implemented in Fstat version 2.9.3 [52].

#### Genetic assignation of WCR sample to parental populations and estimation of the rate of admixture

To determine the degree of admixture of the individuals and their genotypic classes (NW Italy or CSE Europe parental crosses, F_1_, admixed genotypes of 2^nd^, 3^rd^, 4^th^ and 5^th^ generations (because the contact took place 5 generations ago) and backcrosses (Bx)) we sought to assign each individual of each sample to parental populations/outbreaks using the Bayesian method implemented in STRUCTURE version 2.3.3 [53]. For this, we used 6 reference samples from NW Italy and 7 reference samples from CSE Europe outbreaks (Table 1 and Figure 1) as representative samples of parental populations [21]. Because the Veneto region is an admixed zone between two parental populations [21], [44], we performed 20 runs with the number of clusters (*K*) fixed to 2. Each run consisted of a burn-in of 2×10^5^ iterations, followed by 10^6^ iterations. We used the admixture model together with the correlated allele frequencies model [54], without the use of sampling location as prior information [55]. Default values were maintained for all other parameters. Five non-overlapping classes of values of the co-ancestry coefficient *Q* were defined to characterize the individual’s admixed status: (class 1 = [0–0.2]; class 2 = [0.2–0.4]; class 3 = [0.4–0.6]; class 4 = [0.6–0.8]; class 5 = [0.8–1]). As a rule of thumb, classes 1 and 5 should correspond to CSE Europe and NW Italy parental genotypes, respectively; classes 2 and 4 should correspond to backcross genotypes and class 3 should correspond to F_1_ and admixed genotypes of 2^nd^, 3^rd^, 4^th^ and 5^th^ generations. These classes are referred to as Structure classes hereafter. A CLUMPP analysis [56] was performed on the STRUCTURE results to compute similarity indexes between runs and verify that the MCMC did converge.

The coefficient of co-ancestry, *Q,* was also used to compute the individual rate of admixture (ROA) ranging from 0 (100% of the genome is of the NW Italy or the CSE Europe parental type) to 1 (half the genome is of the NW Italy parental type, and the other half is of the CSE Europe parental type). ROA is calculated as follows: ROA = 2*Q* (for *Q*≤0.5) and ROA = 2(1− *Q*) (for *Q*>0.5). ROA is a triangular function of *Q* that is maximal for a *Q* value of 0.5 (pure admixed type) and minimal for a *Q* value of 0 or 1 (parental type).

Therefore the admixed status of each individual was characterized by one qualitative variable obtained using the clustering method (Structure classes), and one quantitative variable (ROA) intimately linked to the Structure classes.

To determine the degree of admixture of the individuals and their genotypic classes we also used the Bayesian genetic clustering approach implemented in NewHybrids 1.1 [57]. This program uses Markov chain Monte Carlo simulations to compute the posterior probability that each individual in a sample falls into six genotypic classes: two parental classes (NW Italy and CSE Europe), F_1_ and F_2_ admixed and two classes of backcrosses (Bx-NW Italy and Bx-CSE Europe corresponding to the offspring of F_1_ and NW Italy matings or F_1_ and CSE Europe matings, respectively). Since our loci are semi-diagnostic [44] it is possible to distinguish easily the 4 admixed classes (F_1_ vs. F_2_ and backcrosses) from each other [57]. The genotypic class of each individual was defined by the class with the largest posterior probability. Because low-frequency alleles were abundant in our dataset, uniform priors for allele frequencies and mixing proportions were used rather than Jeffreys priors as suggested by Anderson [58]. Posterior probabilities were obtained from the mean of 4 runs of 2×10^5^ burn-in iterations followed by 10^6^ million iterations. These classes are referred to as NewHybrids classes hereafter.

### Statistical analysis

#### Impact of the female admixed status on mating probability

To test the hypothesis of a relationship between admixed status of females and their mating status (mated or unmated), we compared the distributions of mated and unmated females among genotypic classes in each sample (Tv11, Tv19 and Vi9) with Fisher’s exact tests on contingency tables.

We also tested the hypothesis of a relationship between the genotypic class of females and their mating status (mated or unmated) with generalized linear mixed models (GLMM) with the mating status as the response variable (two modalities: “mated females” and “unmated females”) with a binomial error distribution, the Structure and NewHybrids genotypic classes as qualitative explanatory variables or the ROA as a quantitative explanatory variable and the sample site as a random explanatory variable:
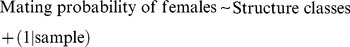
(1)

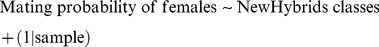
(2)

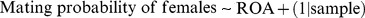
(3)


#### Impact of the male admixed status on mating probability

To test the hypothesis of a relationship between the admixed status of males and their mating probability, we performed the same kind of analyses as above (Fisher’s exact tests and GLMM). However, as mating status of males cannot be determined by direct observation, inferences had to be made using sperm – the genotypes of sperm correspond to the genotypes of mated males. We therefore compared the distributions of sperms (corresponding to mated males only) and sampled males (corresponding to both mated and unmated males) among genotypic classes using Fisher’s exact tests in each sample. In the GLMM, the dependent variable “Mating probability of males” included two modalities, “sperm” and “sampled males”.
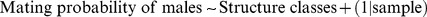
(4)

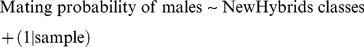
(5)

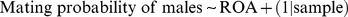
(6)


#### Impact of the admixed status of individuals on survival time

The time between sampling and death under starvation conditions for each of the 600 collected adults was measured. The link between the genotypic class of individuals and their survival time was tested with two mixed Cox’s models [59] with R [60], with the sample as random effect:
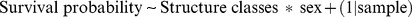
(7)

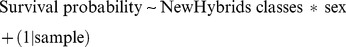
(8)

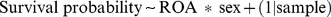
(9)


#### Impact of the genotypic class of WCR individuals on mating choices

We tested the hypothesis of random mating between individuals according to their genotypic class within each sample. For this, we tested the independence between the genotypic class of sperm and that of the female mated with that sperm with Fisher’s exact tests on contingency tables.

## Results

### Determination of sex, dissections of spermathecae and sperm isolation

A total of 327 females and 273 males were collected in Tv11, Tv19 and Vi9 (see Table 2 for more details). Among the 319 genotyped females, 93% were mated (296/319). The proportion of mated females was heterogeneous among fields (*p* = 0.02), with a lower proportion in Tv11 compared to Tv19 and Vi9 (*p* = 0.02 for both comparisons). The number of genotyped males and sperm isolated from within spermathecae was 271 and 190, respectively (Table 2).

### Genetic composition of samples

Overall, the European WCR populations displayed substantial polymorphism, with a mean number of alleles per locus of 6.39 (SD = 4.65), varying from 2.77 in Fontanella, Pince (2010), Szekszard and Budapest (2011) to 4.92 in Tv11, and with a mean expected heterozygosity (*He*) varying from 0.37 for Fontanella and Castegnato (2010) in NW Italy to 0.48 for Tv11 and Vi9 in Veneto (Table 1). *F_IS_* estimates were low and no significant deviation from Hardy-Weinberg equilibrium was observed except in the 3 Veneto samples for which a significant deviation from HW corresponding to a low heterozygote deficit was detected (Table 1). *F_ST_* values between the West and East parental outbreaks are large with a mean value of 0.26 [44].

### Genetic assignment of WCR samples to parental populations and estimation of the rate of admixture

Preliminary Structure analyses were conducted on all samples from Veneto with various values of *K* (from 1 to 4) to confirm the probable existence of only two genetic clusters in the studied area ([44] and results not shown). The method of Evanno [61] pointed to *K* = 2 for each analysis. In addition, analyses with *K*>2 produce incoherent results with samples from NW Italy or CSE Europe being admixed between two clusters instead of being classified as parental samples (data not shown).

**Figure 2:**
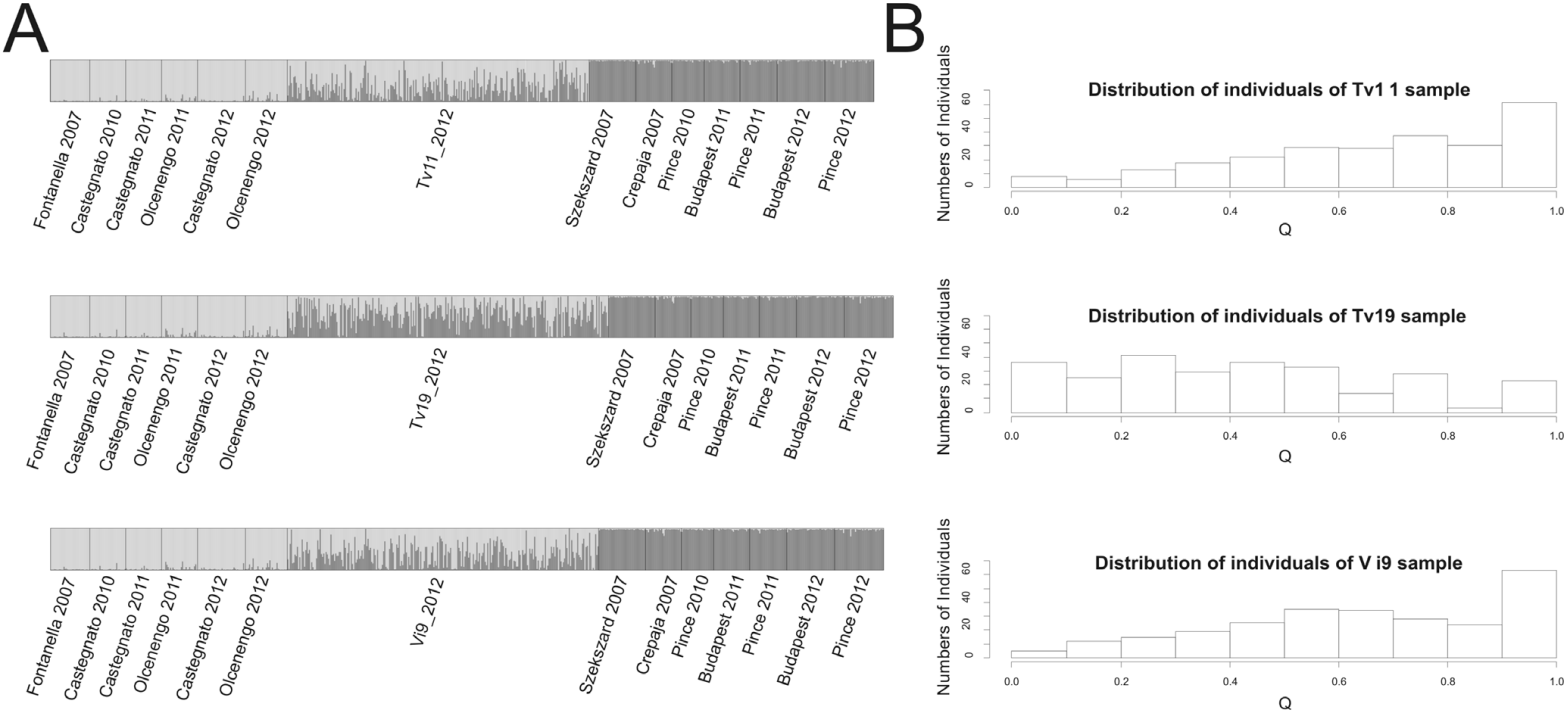
Admixture analysis of Tv11, Tv19 and Vi9 samples resulting from a Structure analysis based on 13 microsatellite loci. The most likely run generated with DISTRUCT version 1.0 is presented here [82]. (A) Assignment of WCR individuals and sperms to *K* = 2 genetically distinct clusters. For each adult or sperm sample, a vertical bar displaying two colours represents the proportion of the genome of the individuals that belongs to each of the two clusters. Adults or sperm samples are grouped by sampling location (the name of which is below the plot). (B) Distribution of the individual coefficient of coancestry *Q* for each sample, Tv11, Tv19 and Vi9.

**Figure 3:**
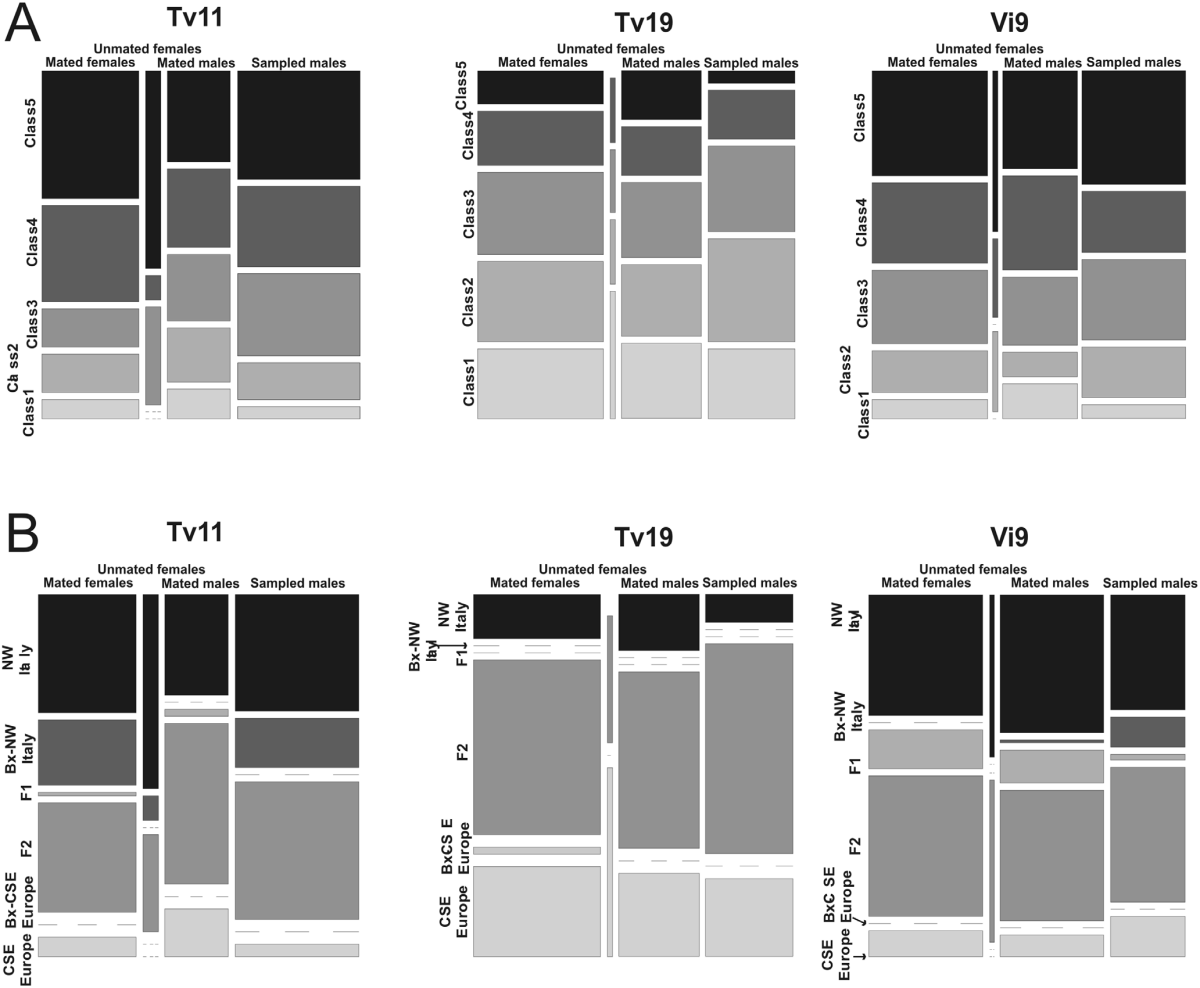
Distribution of the Structure and NewHybrids classes among mated and unmated individuals for each of the sampled fields. (A) Admixed and parental classes distribution (Structure classes) of samples Tv11, Tv19 and Vi9 among mated and unmated females and among mated males (sperm from spermathecae) and sampled males (corresponding to both mated and unmated males). Bx = backcross. Note that the width and the height of Structure classes represent the number of individuals within each of them and their relative frequency, respectively. (B) Admixed and parental classes distribution (NewHybrids classes) of samples Tv11, Tv19 and Vi9 among mated and unmated females and among mated males (sperm from spermathecae) and sampled males (corresponding to both mated and unmated males). Bx = backcross. Note that the width and the height of NewHybrids classes represent the number of individuals within each of them and their relative frequency, respectively.

With *K* = 2, the Structure analysis placed the 6 samples that were representative of the NW Italy outbreak within one cluster (NW Italy, all *Q*>0.8) and the 7 samples that were representative of the CSE Europe outbreak within the other cluster (CSE Europe, all *Q*<0.2), as expected (see Figure 2A). The CLUMPP analyses showed that the similarity among the various runs of the MCMC was larger than 0.99, suggesting a proper convergence of the MCMC. *Q* coefficients of individuals sampled in Tv11, Tv19 and Vi9 vary from 0.014 to 0.989. Overall, 26% of the individuals from these samples were assigned to the NW Italy cluster (with *Q*>0.8), 12% to the CSE Europe cluster (with *Q*<0.2) and 62% were admixed between both clusters (0.2< *Q*<0.8) (Figure 2, Figure 3A and Table 3). For Tv11 and Vi9, a bimodal distribution of *Q* was found with a mode at 1 (a group of individuals is assigned to the NW Italy) and the other mode at 0.8 and 0.6 for Tv11 and Vi9, respectively (Figure 2B). In Tv19, the distribution of *Q* looks like a mixture between a unimodal and a uniform distribution with a flat mode in the interval of 0 to 0.6 (Figure 2B). The five genotypic classes defined by the *Q* values are present in the Veneto region (Table 3, Figure 3A). Their frequency is variable among fields (*p*<10^−3^) with a larger frequency of class 1 and 2, and a lower frequency of class 5 in Tv19 than in Tv11 and Vi9 (*p*<10^−3^ for each comparison). However, the proportion of parental versus admixed classes did not vary significantly among fields (*p* = 0.09 and 0.28 in females and males, respectively).

**Table 3:** Frequency of the different genotypic classes within samples, according to the Structure or NewHybrids analysis.

	Structure-classes					NewHybrids classes					
Fields	Class 1	Class 2	Class 3	Class 4	Class 5	CSE Europe	Bx-CSE Europe	F1	F2	Bx-NW Italy	NW-Italy
Tv11	0.06	0.12	0.20	0.26	0.36	0.07	0.00	0.01	0.42	0.13	0.37
Tv19	0.23	0.26	0.25	0.16	0.10	0.27	0.01	0.00	0.58	0.00	0.13
Vi9	0.07	0.13	0.23	0.24	0.33	0.08	0.00	0.08	0.42	0.03	0.38

The Structure-classes, corresponding to five non-overlapping classes of values of the co-ancestry coefficient *Q* were defined to characterize the individual’s admixed status: class 1 = [0–0.2]; class 2 = [0.2–0.4]; class 3 = [0.4–0.6]; class 4 = [0.6–0.8]; class 5 = [0.8–1]. As a rule of thumb, classes 1 and 5 should correspond to CSE Europe and NW Italy parental genotypes, respectively; classes 2 and 4 should correspond to backcross genotypes and class 3 should correspond to F_1_ and admixed genotypes of 2^nd^, 3^rd^, 4^th^ and 5^th^ generations. NewHybrids classes correspond to six genotypic classes: two parental classes (NW Italy and CSE Europe), F_1_ and F_2_ admixed and two classes of backcrosses (Bx-NW Italy and Bx-CSE Europe corresponding to the offspring of F_1_ and NW Italy matings or F_1_ and CSE Europe matings, respectively).

The analysis of NewHybrids reveals that the majority of expected classes (NW Italy, CSE Europe, F_1_, F_2_, Bx-NW Italy, Bx-CSE Europe) are present in each sample (Figure 3B and Table 3). However, their frequency varies from one sample to another (Fisher’s exact test, *p*<10^−3^). Tv11 and Vi9 have a majority of F_2_ and NW Italy types whereas Tv19 mainly contains F_2_ and CSE Europe types (Figure 3B and Table 3). However, the proportion of parental versus admixed classes does not vary significantly among fields (p = 0.92 and 0.23 in females and males, respectively).

### Influence of admixed status on the fitness of individuals

#### Impact of the female admixed status on mating probability

No tests involving the 5 Structure classes or the 6 NewHybrids classes were significant. Considering the 5 Structure classes and 6 NewHybrids classes led to too few individuals in each class to get a satisfactory statistical power. Thus, in the following, genotypic classes were clustered into 2 modalities: “admixed” and “parental” classes. “Admixed” classes were obtained by pooling Structure classes 2, 3 and 4 and by pooling NewHybrids classes F_1_, F_2,_ Bx NW Italy and Bx CSE Europe. “Parental” classes were obtained by pooling Structure classes 1 and 5 and by pooling NewHybrids classes NW Italy and CSE Europe.

The female mating probability was not significantly influenced by the Structure and NewHybrids classes (Figure 3, GLMM tests, *p* = 0.21 and 0.16, respectively) or by the ROA (GLMM test, *p* = 0.35). Fisher’s exact tests on Structure and NewHybrids classes showed that “parental” and “admixed” classes displayed very similar frequencies among mated and unmated females (Figure 3, *p*>0.05 for all tests). Note that unmated females are probably too few in number (n = 23) to achieve high statistical power. Note also that when the three fields were pooled, no significant tests were obtained.

#### Impact of male admixed status on mating probability

The Structure and NewHybrids classes and the ROA did not statistically influence the mating probability of males whatever the considered variable (Figure 3, GLMM tests, *p* = 0.30, 0.27 and 0.22, respectively). Fisher’s exact test on Structure and NewHybrids classes showed that “parental” and “admixed” classes displayed very similar frequency among mated males (sperm from spermathecae) and sampled males (corresponding to both mated and unmated males) (Figure 3, *p*>0.05 for all tests). Note that when the three fields were pooled, no significant tests were obtained.

#### Impact of the admixed status of individuals on survival time

**Figure 4:**
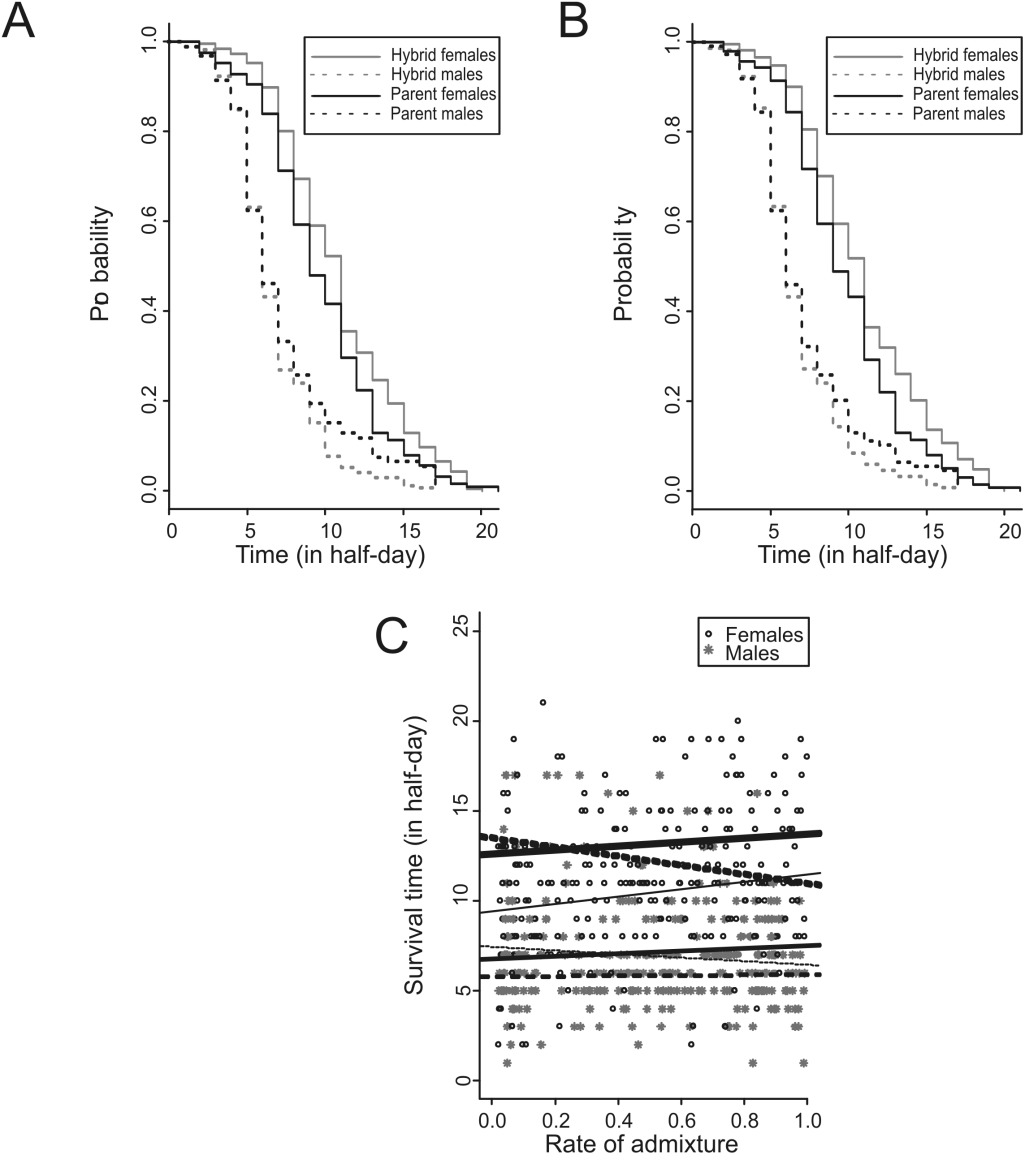
Survival of individuals under starvation conditions as a function of their admixed status. (A) Survival probability of females and males belonging to the two modalities of Structure classes (admixed or parental) as a function of time (mixed Cox’s models *p* = 0.062 and 0.48 for females and males, respectively). (B) Survival probability of females and males belonging to the two modalities of NewHybrids classes (admixed or parental) as a function of time (mixed Cox’s models *p* = 0.048 and 0.69 for females and males, respectively). (C) Survival time of individuals sampled in the population Tv11, Tv19 and Vi9 as a function of their ROA (mixed Cox’s model, *p* = 0.073 and 0.081 for females and males, respectively). Solid line: trend of female survival time. Dashed line: trend of male survival time. Thick lines and very thick lines correspond to a linear regression between survival time and ROA for the first half (0–9 half-days) and the second half (10–20 half-days) of the survival experiment, respectively. Thin lines correspond to the regression made on the entire period.

A significant interaction between sex and Structure classes (*p* = 0.039) and between sex and ROA (*p* = 7.7×10^−3^), and a marginally significant interaction between sex and NewHybrids classes (*p* = 0.073) was found. In females, admixture had a positive and marginally significant impact on survival (*p* = 0.062, 0.048 and 0.073 with Structure and NewHybrid classes and ROA respectively) with admixed individuals suffering a 20% lower mortality hazard than the parents (Figure 4A and 4B). In males, admixture had a negative and non significant effect on survival (*p* = 0.48, 0.69 and 0.081 with Structure and NewHybrids classes and ROA respectively) (Figure 4). Note that the interactions between sex and Structure classes, NewHybrids classes and ROA were significant in Vi9 (*p* = 0.016, 0.025, 0.005, respectively) but not in Tv11 and Tv19 (*p*>0.3 for all 6 tests). The effect of the interaction between admixed status and sex was mostly observable in the second half of the survival experiment with Structure classes and ROA (*p* = 0.026, 0.15 and 0.023, with Structure and NewHybrids classes and ROA, respectively) with a larger mortality hazard in admixed than in parents in males (*p* = 0.03, 0.22 and 0.036 for Structure and NewHybrids classes and ROA, respectively). Again this was true in Vi9 only (effect of the interaction between admixed status and sex, *p* = 0.010, 0.036, and 0.017; and larger mortality hazard in admixed than in parents in males, *p* = 0.028, 0.15 and 0.03 with Structure and NewHybrids classes and ROA, respectively).

The global effect of sex on the mortality hazard was large and significant (*p*<10^−5^). It was about 4 and 2.5 times larger in males than in females in admixed and parents, respectively (Figure 4).

#### Impact of the genotypic class of WCR individuals on mating choices

Fisher’s exact tests indicated that, in all samples, the genotypic classes (“admixed” or “parental” determined from the Structure or NewHybrids analyses) of sperm and that of the female mated with that sperm were not statistically dependent on each other (*p*>0.05 for all comparisons). Therefore, no choice of the sexual partner according to its admixed status has been detected. Note that when the three fields were pooled, no significant tests were obtained.

## Discussion

In this study, we tested whether admixed status influenced individual fitness in the context of the European invasion of WCR. Our results show that mating probability and the choice of sexual partners are not influenced by their admixed status. However, the admixed status of the adults has a non-straightforward effect on adult survival under starvation conditions. This effect is sex- and field- dependent and is mostly observable in later dying adults.

### Admixed individuals are common in the centre of the WCR contact zone

Under the hypothesis of a selection against admixed individuals, frequencies of parental genotypes should be higher than frequencies of admixed genotypes in an admixed zone [62]. As a consequence, the distribution of the *Q* coefficients is expected to be U-shaped, with numerous low (0–0.2) and high (0.8–1) *Q* values and relatively few intermediate (0.2–0.8) *Q* values. This is not what we observed: (i) results of Structure analyses show that the 3 samples contain a majority of admixed individuals; (ii) Tv11 and Vi9 contain very few individuals belonging to the class 1 (0–0.2), probably because the samples were collected in the western part of the contact zone rather that in its centre.

### Admixed status does not clearly influence individual fitness

In this study, we sought to determine whether the genotypic classes (Structure and NewHybrids classes) and the ROA of individuals influenced their probability of mating and their ability to survive to stressful conditions. To do this, we used statistical tests (Fisher’s exact tests, GLMM and mixed Cox’s models) (i) to compare the genotypes of mated females with those of unmated females, (ii) to compare the genotypes of all males with those of successful mated males, *via* the genotype of sperm contained in mated females, (iii) to test whether mating is random according to genotypic classes, and (iv) to test if the genotypic classes and the ROA had an impact on the survival time of adults under starvation.

Concerning the comparisons of the genotypes of mated versus unmated females and of all males versus mated males, no significant admixture effect was detected. We therefore conclude that, overall, the admixed status of males and females did not influence their mating probability. The absence of a significant effect is likely not due to statistical power issues as pooling individuals from the three fields did not result in significant relationships between genotypic classes and mating probability.

Regarding the random mating hypothesis, no association was detected between the genotypic classes of males and that of females within mated couples: the genotypic classes of the mated females and that of the sperm in the females were not statistically associated. Again, statistical power issues are likely not the cause of the absence of an effect as pooling the three fields has no effect on the tests’ significance.

Concerning the analyses of survival using Cox’s models, our result show that sex had an effect on survival, with the average mortality hazard about 3 times larger in males than in females. This result was unexpected because the literature suggests that WCR males live longer than females *in natura* (102 days for males against 78 for females [63]). Two hypotheses may explain this result: (i) our samples were collected after mating of the majority of females, at a time when they are feeding while males are looking for sexual partners and have probably fewer resources than females [42], [63]. Given that the survival test took place under starvation conditions, males have probably suffered a disadvantage compared to females; (ii) at the time of mating, the males transfer their sperm to the females *via* the spermatophore. In some insects, the spermatophore is associated to the spermatophylax, a nutritive vesicle given by males to females as nuptial gift that extends the mating time and ensures the sperm transfer [64], [65]. This nutritive vesicle is present in WCR [66] and can constitute a significant portion of male mass [67]. If females also use the spermatophylax as a nutritive resource, they may benefit energetically relative to the males, explaining their greater survival.

Analyses of survival using Cox’s models also show a sex-specific effect of the admixed status on survival under starvation conditions. Admixed females suffer a 20% lower mortality hazard than parental females and admixed males have a larger mortality rate than parental males, mostly in the second half of the experiment period (the latest 5 days). The positive effect of admixture in females may be the consequence of the fixation of deleterious mutations in each parental invasive outbreak [26], [32], [68]. The effect in males – outbreeding depression – was not expected [44] because the isolation time between both parental outbreaks was probably short (about 20 generations) for Dobzansky-Müller or breaking down of co-adapted gene complexes effect to occur [69], [70]. We do not have any satisfactory explanation for the difference between sexes.

To what extent is such an effect of admixture on adult survival biologically significant *in natura*? First, we believe that WCR starvation conditions are uncommon in the field, particularly in an area of intensive maize production. WCR adults eat maize foliage, silks, pollen and even developing kernels [71], [72]. WCR populations begin to emerge at a time when they can readily feed on maize foliage while it is still in vegetative stage of development. WCR adults continue to feed on silks, pollen and developing kernels as the plants mature. Some moist silks and accessible kernels are available to WCR well into the dent stage [73] making it possible for WCR to feed on maize tissues for more than a month after completion of silking [73]. Only late in the season, once maize is mature, may WCR be unable to find suitable maize tissues to feed on. However these late survivors are able to eat pollen from various plants including weeds [74]. Second, a difference in survival between genotypes would be biologically significant *in natura* only if it took place before reproduction. Our results show that most of the difference in adult male survival took place during the second half of the test period. Although we did not know the age of the individuals at the time of their death, this suggests a moderate impact of the survival difference on fitness. Third, the effect is significant in only one of the three fields sampled and therefore may depend on the environment. Fourth, the effect is sex-specific with opposite signs in each sex. This suggests that a putative fitness cost of admixture in one sex could be, at least partially, compensated by a fitness gain in the other sex. Overall, we hence believe that the complex effect of admixture on adult survival may not be relevant *in natura*.

The absence of a clear effect of admixture on WCR fitness components might be explained by the timing of the study within the invasion process. Indeed, most studies that revealed a positive impact of admixture on invasion were conducted in situations where admixture occurred in the first steps of invasions, soon after introductions [22], [32], [75]. In the present study, our sampling and tests occur about 12 and 20 generations after the introductions of WCR into the two invaded European areas. Impacts of admixture might greatly differ between early contacts soon after primary introductions and secondary contacts. Assuming that the invasion success partly depends on the capacity of a population to respond to new selective pressures, in cases of secondary contacts such as observed in Veneto, both populations may have already proved successful and may have evolved towards greater fitness. Hence, it may be more difficult to detect positive effects of admixture in this context.

It is also possible that an impact of the admixed status of the individuals on their fitness exists but was not clearly detected because of the choice of the traits measured in this study. Here we measured two traits related to fitness – the mating rate and the survival time of adults under starvation – that can hardly be generalized to all components of fitness. Other traits, often used in similar studies, like body parameters (size or dry mass) or realized fecundity or flight activity [76]–[78] may have been a better choice to detect a fitness difference between genotypes. An absence of a significant effect is difficult to generalize into the absence of an effect. It is noteworthy that unpublished results revealed no effect of admixture on other phenotypic traits (see next section, Stefan Toepfer, HongMei Li, Gerald Bermond, unpublished data) and confirmed the present results.

### Neutral contact zones to estimate dispersal

Recently, Bermond et al. [44] used the admixed zone of Veneto to estimate dispersal capabilities of WCR by studying the geographic clines in allelic frequencies of microsatellite markers. The dispersal of WCR was estimated at about 20 km/generation. Bermond et al. [44] hypothesized that the size of the admixed zone (the width of the clines) increases at a rate that depends on WCR dispersal. This estimate relies on the idea that the admixed zone is neutral, *i.e.* (i) individuals of parental and admixed types do not differ in fitness and (ii) mating is random.

Previous laboratory tests on phenotypic traits of WCR did not show any significant difference in measured traits between parental (NW Italy and CSE Europe) strains and F1 crosses (Stefan Toepfer, HongMei Li, Gerald Bermond, unpublished data). The observed phenotypic traits were individual weight, length and width of the elytra, fecundity, egg survival during the winter, and survival of larvae and pupae. The average value of phenotypic traits of admixed populations was either an intermediate value between the values of the two parental populations or equal to that of one of the parents. These results are globally consistent with those of the present study. Considering all the traits analysed here, our results suggest that the contact area is probably close to neutrality and that mating is random. Our findings are wholly consistent with the hypothesis made by Bermond et al. [44]. It is noteworthy that a new analysis of the geographic clines of allelic microsatellite frequencies in the next years will allow the hypothesis of a neutral admixture zone to be definitively tested. Temporal stability of the clines - with a constant width through time - will clearly favour a tension zone characterized by a lower fitness of the admixed individuals [79], [80]. On the contrary, if the clines vanish through time, a neutral admixture zone or a zone in which admixed individuals experience a fitness gain will be favoured.

### Conclusion

In the present study, most results on fitness were obtained without manipulation or experimentations and were directly obtained from individuals collected *in natura*. In addition, the results we obtained are nuanced: despite a global absence of and effect of the admixed status on traits related to fitness, a sex- and location- dependent effect of admixture was noticed in the laboratory for a single trait (survival under starving conditions). Whether admixture generally has a positive impact on invasion remains to be carefully studied and more case studies on ongoing invasions are necessary to reach definitive conclusions [11], [20], [81]. Current invasions constitute many field models on which invasion biologists can test the role of admixture, keeping in mind the critical importance of negative results in the literature.

## Supporting Information

Figure S1 **(A) Dissected spermatheca of** ***D. v. virgifera*** **. (B) Enlargement of spermatozoa present in spermatheca. SP: spermatheca; SPZ: spermatozoa.**
(TIF)
