# Supplementary figures and images for: No Clear Effect of Admixture between Two European Invading Outbreaks of Diabrotica virgifera virgifera in Natura
Source: PLoS One. 2014 Aug 29;9(8):e106139. doi: 10.1371/journal.pone.0106139 (PMC4149517; doi:10.1371/journal.pone.0106139)

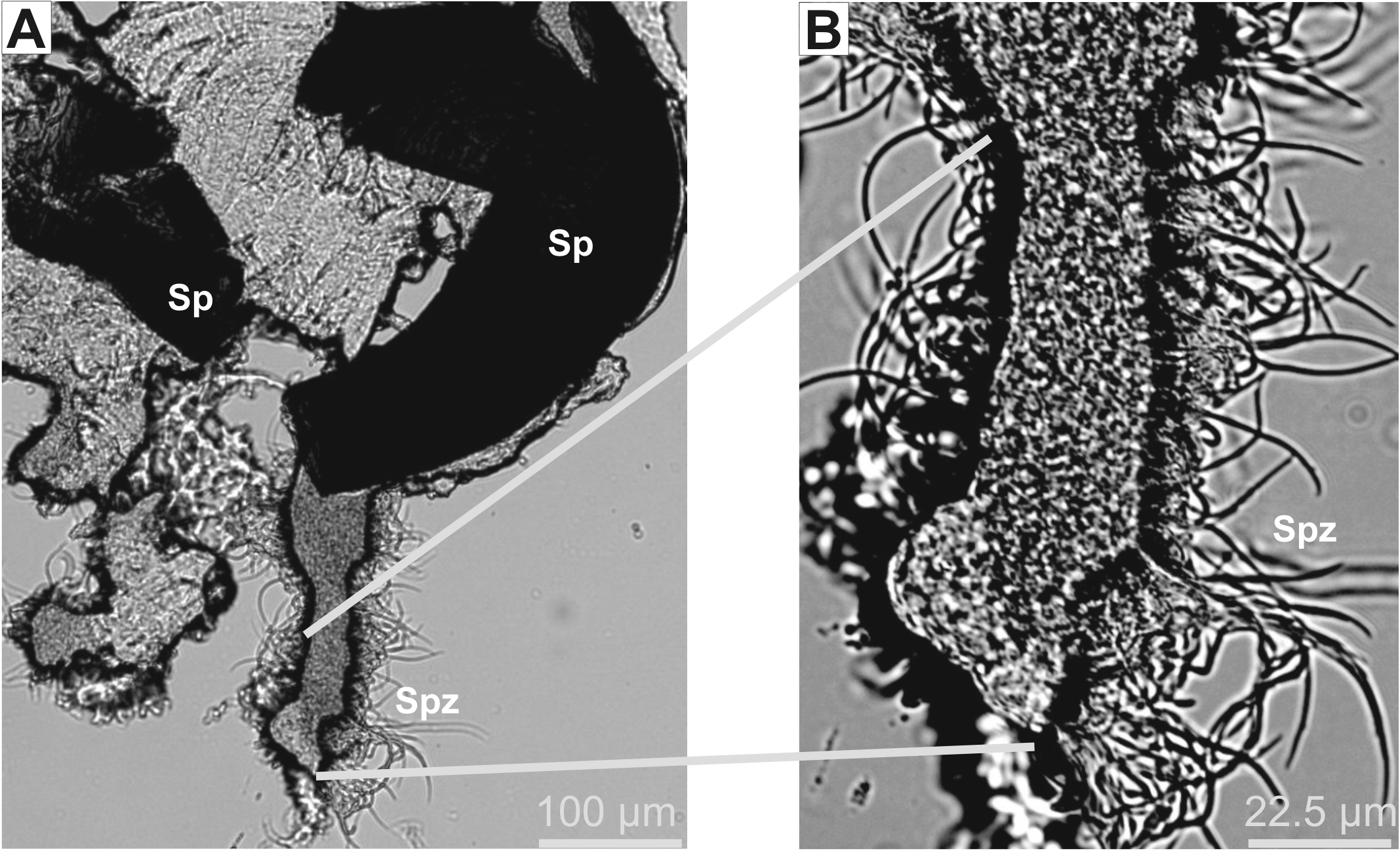

Supplement: Figure S1 — (A) Dissected spermatheca of D. v. virgifera . (B) Enlargement of spermatozoa present in spermatheca. SP: spermatheca; SPZ: spermatozoa. (TIF) [file pone.0106139.s001.tif]
